# Identifying Electronic Nicotine Delivery System Brands and Flavors on Instagram: Natural Language Processing Analysis

**DOI:** 10.2196/30257

**Published:** 2022-01-18

**Authors:** Rob Chew, Michael Wenger, Jamie Guillory, James Nonnemaker, Annice Kim

**Affiliations:** 1 Center for Data Science RTI International Research Triangle Park, NC United States; 2 Center for Health Analytics, Media, and Policy RTI International Research Triangle Park, NC United States

**Keywords:** named entity recognition, ENDS, social media, brands, flavors

## Abstract

**Background:**

Electronic nicotine delivery system (ENDS) brands, such as JUUL, used social media as a key component of their marketing strategy, which led to massive sales growth from 2015 to 2018. During this time, ENDS use rapidly increased among youths and young adults, with flavored products being particularly popular among these groups.

**Objective:**

The aim of our study is to develop a named entity recognition (NER) model to identify potential emerging vaping brands and flavors from Instagram post text. NER is a natural language processing task for identifying specific types of words (entities) in text based on the characteristics of the entity and surrounding words.

**Methods:**

NER models were trained on a labeled data set of 2272 Instagram posts coded for ENDS brands and flavors. We compared three types of NER models—conditional random fields, a residual convolutional neural network, and a fine-tuned distilled bidirectional encoder representations from transformers (FTDB) network—to identify brands and flavors in Instagram posts with key model outcomes of precision, recall, and F1 scores. We used data from Nielsen scanner sales and Wikipedia to create benchmark dictionaries to determine whether brands from established ENDS brand and flavor lists were mentioned in the Instagram posts in our sample. To prevent overfitting, we performed 5-fold cross-validation and reported the mean and SD of the model validation metrics across the folds.

**Results:**

For brands, the residual convolutional neural network exhibited the highest mean precision (0.797, SD 0.084), and the FTDB exhibited the highest mean recall (0.869, SD 0.103). For flavors, the FTDB exhibited both the highest mean precision (0.860, SD 0.055) and recall (0.801, SD 0.091). All NER models outperformed the benchmark brand and flavor dictionary look-ups on mean precision, recall, and F1. Comparing between the benchmark brand lists, the larger Wikipedia list outperformed the Nielsen list in both precision and recall.

**Conclusions:**

Our findings suggest that NER models correctly identified ENDS brands and flavors in Instagram posts at rates competitive with, or better than, others in the published literature. Brands identified during manual annotation showed little overlap with those in Nielsen scanner data, suggesting that NER models may capture emerging brands with limited sales and distribution. NER models address the challenges of manual brand identification and can be used to support future infodemiology and infoveillance studies. Brands identified on social media should be cross-validated with Nielsen and other data sources to differentiate emerging brands that have become established from those with limited sales and distribution.

## Introduction

### Background

Social media platforms provide opportunities for brands to market products to users and potential users of tobacco products [1-3]. JUUL was one of the first electronic nicotine delivery system (ENDS) brands to engage in extensive social media marketing on Instagram, Facebook, and Twitter starting in 2015, with the marketing prominently featuring sweet and fruit-flavored products (eg, mango) [4]. JUUL, to some extent, took the tobacco control community by surprise. From the company’s inception, JUUL sales grew rapidly, with the company contributing substantially to the 97% growth of the ENDS marketplace between 2015 and 2018 [5,6].

Social media marketing of ENDS products has become widespread, a study conducted by the Campaign for Tobacco-Free Kids and Netnografica identifying >100 social media marketing campaigns conducted by tobacco industry giants via paid social media influencers, which elicited 8.8 billion views in the United States and 25 billion views worldwide [1]. Unsurprisingly, more than half of the youths said that they had recently seen vaping advertisements and that social media was the primary avenue of exposure [2].

Social media marketing of ENDS products coincides with the increasing prevalence of ENDS use among youths and young adults [7-11]. In 2011, current ENDS use (ie, past 30-day use) was 1.5% and 4.9% among high schoolers and middle schoolers, respectively [7], whereas, in 2019, ENDS use was 27.5% and 10.5% among high school and middle schoolers [8]. In 2013 and 2014, current ENDS use was 12.5% among young adults aged 18 to 24 years [9], whereas in 2019, ENDS use was 24.5% among the same age group [10]. Current ENDS use and JUUL use are also higher among young adults (under 24 years) than use among older adults (aged ≥25 years) [11,12]. A recent longitudinal cohort study also showed that marketing ENDS products (via web-based advertising broadcast, radio or internet radio, retail advertising, and billboards) to youths who never used ENDS products predicted ENDS initiation in young adulthood [13], establishing a link between ENDS marketing and use.

ENDS are available in a myriad of flavors (eg, fruit, dessert, mint, and menthol) that appeal to youths and young adults [14,15]. Indeed, 22% of youth ENDS users said that they used ENDS as “they are available in flavors, such as mint, candy, fruit, or chocolate,” and 69% of current youth ENDS users report the use of flavored ENDS [15]. More than three-quarters of young adult ENDS users said that the first ENDS product they used was flavored [14].

The connection between social media marketing and increasing rates of ENDS use, particularly flavored ENDS, among youths and young adults underscores the importance of being able to identify emerging brands and flavors to inform tobacco control research and policy. Previous studies have typically identified brands on the web using manual searches or web scraping. For example, Zhu et al [16] identified 466 brands by searching using multiple search engines and then checking websites. Hsu et al [17], in 2016 to 2017, searched the same brand websites as identified by Zhu et al [16] and found that only 288 of the 466 brands were still active and identified 145 new brands. O’Brien et al [18] also used a manual search strategy of multiple data sources to identify tobacco product brands. These studies suggest that vaping brands are continually emerging, and a more scalable, automated method is needed to identify emerging vaping brands and flavors to inform public health surveillance.

Several recent studies have used computational models to characterize ENDS brands and flavors in vaping-related social media posts. Vandewater et al [19] identified posts from 4 brands and then used text mining to differentiate brand posts (ie, to predict which of 4 brands posted a message). Measures included length of post (eg, Blu had the shortest posts) and words used (eg, Blu and NJOY used lifestyle words, and Logic and Metro focused on product purchase, device, and use). This study showed that computational text mining could help identify and predict linguistic patterns in posts by brand. Xie et al [20] developed named entity recognition (NER) models for several entity types using text from an e-cigarette forum. NER is a natural language processing task that identifies certain words (entities) within text based on the characteristics of the entity and its surrounding words. The models were trained on a labeled data set in which specific entities were manually labeled. e‑Liquid flavors were the most difficult type of entity for models to consistently identify, with F1 scores ranging from 0.0 to 0.786 across models. These studies demonstrate the potential benefit of using computational methods to analyze large volumes of social media data at scale to identify and predict patterns more quickly than traditional manual methods. To date, computational methods have not been used to identify emerging vaping brands, and limited work has demonstrated the benefit of computational methods to identify flavor mentions on social media [20].

### This Study

In this study, we develop NER models to identify potential emerging vaping brands and flavors on Instagram. In our adaptation, the entities of interest are brands and flavors, and the training data comprises manually labeled brands and flavors in the Instagram post text. A benefit of using NER for brand and flavor identification is that a well-performing model will not only identify whether a post contains brand or flavor mentions but can also identify what the brand or flavor is and where in the post the brand or flavor mention occurs. Quantifying how often certain brands and flavors are mentioned can not only help in understanding the growth in vaping marketing discussions generally but may also help identify new emerging brands on social media in a systematic way.

## Methods

### Data Collection

Our data set included 2272 Instagram posts that were manually coded (Table 1). We used Brandwatch, a commercial social media monitoring platform, to extract all Instagram posts that appeared in the query from April 22 to April 23, 2020, with mentions related to vaping (eg, #vape, #ecigs, #vapelife, #vapecommunity) and excluding mentions of cannabis and cannabidiol (eg, #cannabis, #cbd, #420), for use in manual coding (see Multimedia Appendix 1 for the full query). We coded the first 2272 posts from this period.

**Table 1:** Statistics of annotated data.

Data item	Frequency
Posts	2272
Tokens	79,401
Unique tokens	11,102
Brand mentions	1235
Flavor mentions	506

#### Data Annotation

**Figure 1:**
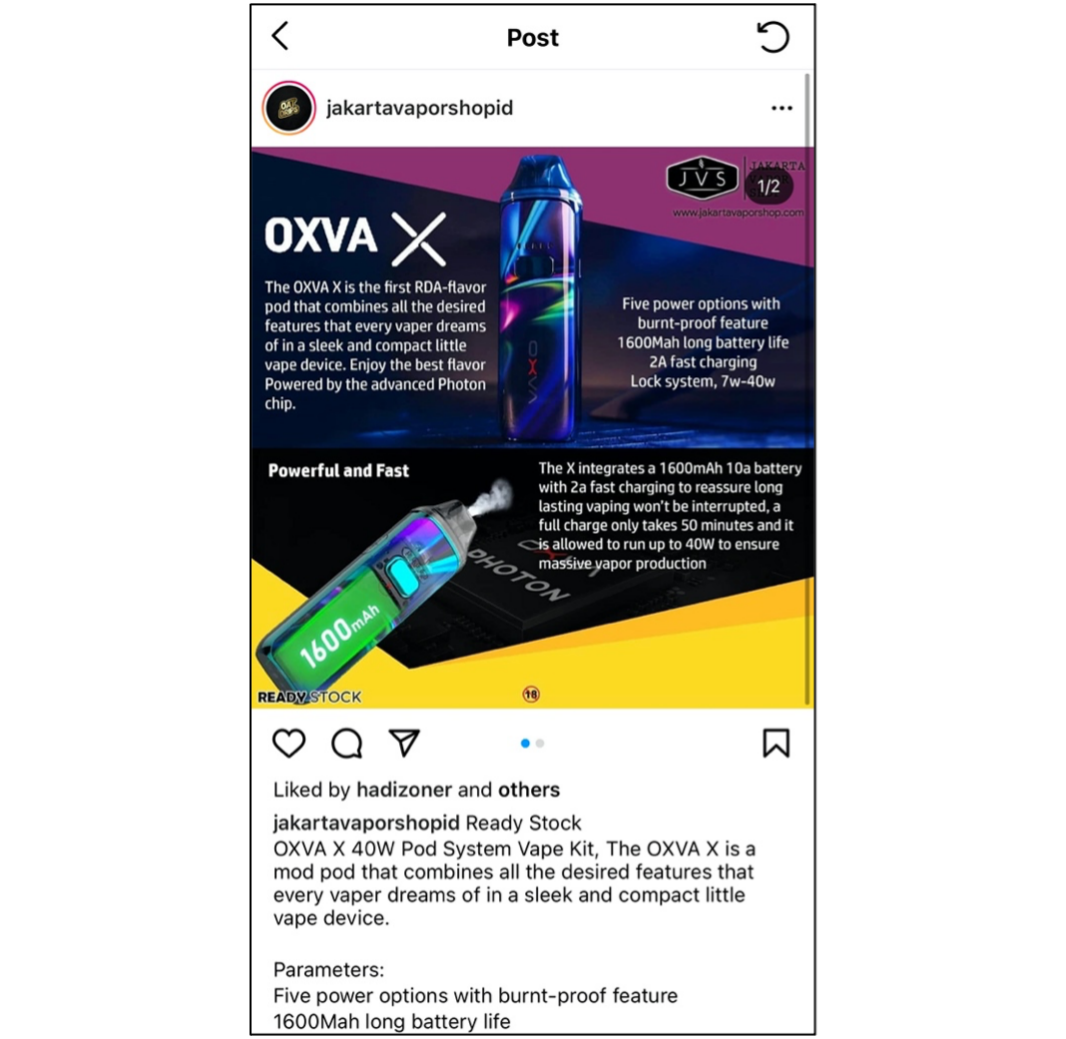
Example of a vaping post on Instagram.

We trained 2 coders to manually label posts to identify mentions of ENDS brands and flavors (see example post in Figure 1). Coders were instructed to exclude posts from coding if the post (1) did not mention a vaping brand, (2) focused on cannabis or cannabidiol products, or (3) was in a language other than English. For the purposes of brand identification and coding, we defined a brand as an ENDS product that was manufactured and sold under a specified name, which we distinguished from specified product lines (ie, specific groups of products sold under a single brand name). For example, Puff Bar is a brand name that sells several product lines, such as Puff Bar, Puff XXL, and Puff Plus. Coders also coded posts for mentions of flavors, with our definition including both common flavors that describe fruit and dessert (eg, raspberry and crème brulee) and flavors with ambiguous flavor names (eg, tropical and blue). Coders also used data on brands and flavors from other data sources (eg, Nielsen scanner data and search engine queries) to inform the coding of brands.

**Figure 2:**
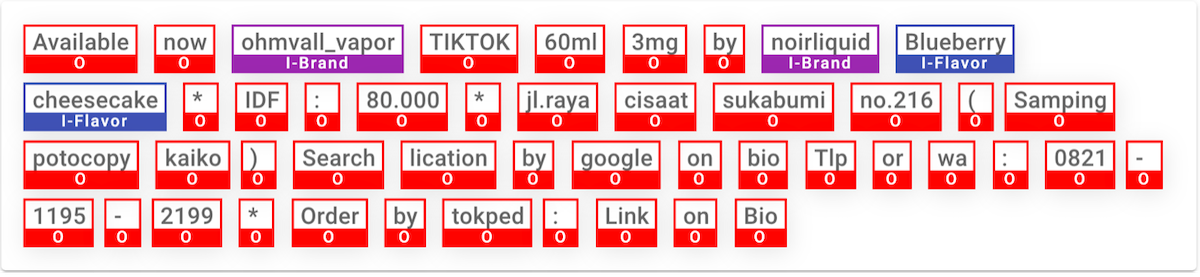
Example of annotated post text after inside–outside encoding was applied.

Coders were provided with a codebook that included the above definitions for ENDS brands and flavors with example brand and flavor names. During a preliminary training session, we showed coders a series of example Instagram posts where they were asked to identify brands and flavors in the text of posts and received feedback from a third adjudicator. To facilitate coders’ annotation of the Instagram post text, we loaded Instagram post text and links to Instagram posts into a custom web-based user interface that coders accessed with a personalized username and password. Coders used this interface to annotate ENDS brands and flavors in Instagram post text by highlighting the relevant post text and using a dropdown menu to identify the highlighted text as *brand* or *flavor*. Coders were instructed to open the provided Instagram post link if additional contextual information was needed to annotate post text with ENDS brands and flavors (see Figure 2 for an example of annotated post text). During the training period, coders double coded sets of 50 test posts, and interrater reliability was assessed using Cohen *κ*. Once we achieved interrater reliability of Cohen *κ*>0.70 (brands: 0.95; flavors: 0.74), coders single coded the final sample of 2272 posts, with each coder single coding approximately half of the posts.

To prepare the data for modeling, we segmented the posts into tokens, delineated by special characters such as white space or punctuation, using the tokenization required for each model [21,22]. Hashtags were removed from the post text, and emojis were replaced by their Unicode common locale data repository text short names. The inside–outside (IO) encoding scheme was used for its simplicity and as it performed comparably with the other encoding schemes tested, including the IO-beginning and beginning-inside-last-outside-unit schemes. Figure 2 provides an example of the IO encoding scheme applied to an ENDS Instagram post; items classified as O represent text that is outside of the labeled text (brand and flavor).

### NER Models and Statistical Analysis

#### NER Models

#### Conditional Random Fields

Conditional random fields (CRFs) [23] are graphical models commonly used for NER. Although NER can be performed as a traditional classification task, where attributes of an observation are used to infer a categorical designation and each observation is treated independently, graphical models such as CRF acknowledge that text and other structured data often exhibit strong conditional dependence between neighboring observations (ie, neighboring words). This class of models predicts named entities based on an entire sentence rather than making isolated, individual predictions for every word that are not informed by local contextual information.

CRFs model the dependencies between words as an undirected graph between labels and word attributes. These attributes were encoded by the research team as feature functions, which encapsulate the characteristics of a word that may be useful for discriminating between entity types. Textbox 1 summarizes the feature functions used as input variables for the CRF model that were calculated for each token in the training set. Feature functions can also include the characteristics of neighboring words, both before and after the current token. This set of functions represents common feature functions that have been used in other NER brand extraction models [24,25] and NER models in the vaping domain [20].

Specifically, we used a linear chain CRF, which can be thought of as a sequential extension of logistic regression [26]. To learn the model weights, we used the limited-memory Broyden–Fletcher–Goldfarb–Shannon (BFGS) constrained optimization method for gradient descent [27] with an elastic net regularization [28] condition added to the loss function. The CRF model was developed using the sklearn-crfsuite library, a Python wrapper of the popular CRFsuite software (version 0.12; Okazaki) [29].

**Conditional random field feature functions.**

**Conditional random field feature functions**

- Current token is lower cased
- Current token is upper cased
- Current token is title cased
- Current token is a digit
- Prefix of current token
- Suffix of current token
- Previous token is lower cased
- Previous token is upper cased
- Previous token is title cased
- Next token is lower cased
- Next token is upper cased
- Next token is title cased

#### Residual Convolutional Neural Network

As an alternative to CRFs, we trained a residual convolutional neural network (RCNN) using the spaCy Python library [30]. Similar to the development of feature functions in the CRF, we created the (1) lower cased version of the token, (2) token prefix, (3) token suffix, and (4) token shape to extract potentially noteworthy features for each token in the training set. To incorporate these components into a numerical representation, each of the components was individually hashed using a Bloom filter, and the hashes were combined to create a probabilistically distinct vector [31]. In addition to this strategy being memory efficient, it also handles out-of-vocabulary tokens better than traditional word embedding approaches such as word2vec [32] as it incorporates subword information. This embedding was fed forward to a maxout layer [33] to learn a piecewise linear activation function over the inputs.

To add the context of neighboring words, the current token embedding was concatenated to the embeddings from neighboring tokens, fed to another maxout layer, and run through several residual connections [34]. A final step to consolidate the state across words was performed, which was then fed to a softmax layer to return predicted probabilities across entity types. The implementation for this study was trained from scratch using randomly initialized weights. Dropout [35] was used for regularization to help prevent overfitting, and a compounding minibatch strategy was used to increase the batch size throughout the iteration [36]. The Adam optimization algorithm [37] was used to learn the weights over 50 iterations.

#### Fine-tuned Distilled Bidirectional Encoder Representations From Transformers

For the final NER model, we used transfer learning to fine-tuned distilled bidirectional encoder representations from transformers (FTDB) model to our ENDS brand and flavor NER tasks using the HuggingFace Transformers library [38] in Python. Transfer learning [39] is a machine learning framework in which the objective is to use a model trained in one source domain or task to develop a model in a related target domain or task. When implemented effectively, transfer learning can provide useful representations learned in the source domain to help accelerate learning and require less labeled data in the target domain. We performed a method of transfer learning used in deep learning models called fine-tuning [40]. We started with a network model that has been trained in a source domain on a specific task, or set of tasks, and used these weights to initialize a model that is updated for a task in the target domain (eg, learning to identify ENDS brands and flavors in Instagram post text). Traditionally, model weights are initialized by assuming little knowledge about what the appropriate starting values for the weights should be. For example, random weight initialization was used to train the RCNN model in this study. Starting with pretrained weights rather than random initializations allowed the model to initiate with components that benefit the new target task and are often challenging to learn anew with limited training data.

DistilBERT [22] is a deep neural network derived from the bidirectional encoder representations from transformers (BERT) architecture [41]. BERT is a transformer model [42], a type of neural network that takes a sequence of tokens as input and produces a contextualized vector representation of each token as its output, primarily using a mechanism called attention. BERT uses multiheaded attention layers to dynamically assign a weight to every pair of words in a sequence, where the weight indicates how much attention the model should pay to the first token when computing the representation of the second token. The multiple attention heads within each layer are trained in parallel and can each potentially capture different word–word relations. For example, Manning et al [43] showed that attention heads in BERT learned foundational linguistic characteristics such as syntactic grammatical relationships and anaphoric coreference, despite not being trained for those objectives.

BERT is pretrained on two supervised learning tasks in which labels are already encoded in the text and do not require additional manual annotation: (1) masked language modeling, which replaces random tokens in a sequence with a special mask symbol and training a model to predict the premasked token from the surrounding context, and (2) next sentence prediction, which trains a model to predict whether the next sentence is probable, given the current sentence. BERT was trained on BooksCorpus (800 million words) [44] and English Wikipedia (2500 million words), allowing it to learn masked language modeling and next sentence prediction on a massive amount of written English text. Learning these tasks on such a large corpus allowed fine-tuned BERT models to reach state-of-the-art performance on a variety of natural language understanding tasks such as question answering, semantic textual similarity, and sentiment analysis [41]. However, fine-tuning and making new predictions with BERT is still relatively resource intensive because of the number of model parameters (110 million).

DistilBERT is a compact model trained to mimic BERT that is 40% smaller and 60% faster when creating new predictions while performing similarly [22]. The model compression was achieved by using a method called knowledge distillation [45,46], in which a *student* model (distilled BERT [distilBERT]) is trained to reproduce the behavior of a larger *teacher* model (BERT), in this case, by training distilBERT to predict the BERT’s output class probabilities. Our NER model fine-tunes the distilBERT weights by training it on the task of ENDS brand and flavor token recognition using labeled data from Instagram posts. During training, we used a batch size of 16, a weight decay of 0.01, and 500 warm-up steps over the course of 3 epochs.

#### Benchmarks

#### Overview

As a comparison to the NER models, we created benchmarks to check if brands from an established ENDS brand list are mentioned in the Instagram posts. This approach helped us to better understand coverage gaps between brands mentioned on social media versus established brand lists and helped to assess whether NER modeling aids in emerging brand detection. Although not created for cataloging ENDS brands and flavors on social media, these benchmarks represent data sets that researchers may naturally be inclined to use for ENDS brand and flavor identification on social media, because of their popularity or availability, or as a reference when developing their own databases.

To determine if a post contained a brand from an existing list, we performed a dictionary look-up of the brand or flavor names within the post text. Any mention of the brand or flavor name within the post text was flagged as a potential mention, favoring higher recall over precision. In addition, we compared the top-selling brands in the Nielsen Retail scanner data set with the most mentioned brands in our hand-labeled Instagram data to better understand differences in brand composition.

#### ENDS Brands and Flavors From Nielsen Retail Scanner Data Set

The Nielsen scanner data set for ENDS brands (N=66) is a unique list of brands derived from any sales of ENDS products in large food and convenience stores in the United States from March 22, 2020, to May 16, 2020, a span that overlaps with the dates of the Instagram posts in our data set. These data also include information on ENDS flavors (N=219) sold over the same period.

#### ENDS Brands From Wikipedia Data

The Wikipedia data set for ENDS brands (N=249) is a publicly available, crowdsourced list of electronic cigarettes and vaping liquid brands [47]. Although less informative as a ground-truth source of ENDS sales data, the Wikipedia list is larger, providing a potentially more comprehensive and diverse list of ENDS brands than the Nielsen scanner data set, which focuses on top-selling brands. Wikipedia entries and lists have been used and repurposed in various studies [48,49]. The version of the list used in this study was accessed on March 16, 2020.

#### Validation

#### Overview

To prevent overfitting, we used a 5-fold cross-validation methodology. Cross-validation requires creating models on different, mutually exclusive partitions of training and validation observations. For 5-fold cross-validation, 5 different models (using the same model type) were created and assessed on 5 nonoverlapping validation sets. By noting differences in model performance across the runs, we can gain a more realistic range of results for how a model will generalize on new samples, assuming that the samples are drawn from the same underlying distribution as the training data.

We used the standard evaluation metrics to assess different aspects of the predictions. All metrics are bounded between 0 and 1, and, all else being equal, a higher value indicates a better performing model.

#### Precision

Precision is the fraction of true positives out of all observations that are predicted to be positive.

Precision = true positive / (true positive + false positive) **(1)**

#### Recall

Recall is the fraction of true positives detected out of all positive labeled examples.

>Recall = true positive / (true positive + false negative) **(2)**

#### F1

This summary metric balances the need to find positive examples with the need to reduce the number of false positives.

F1 = 2 / ((1 / recall) + (1 / precision)) **(3)**

## Results

### Brands

Table 2 summarizes the NER and benchmark dictionary look-up approaches across a 5-fold cross-validation scheme. For brands, the mean precision was higher than the mean recall across all methods except for the FTDB, ranging from 0.082 to 0.797 for mean precision and 0.022 to 0.869 for mean recall. The RCNN exhibited the highest mean precision (0.797, SD 0.084) for brands, and the FTDB exhibited the highest mean recall (0.869, SD 0.103). However, despite the FTDB exhibiting the highest mean recall, it also had the largest SD in recall scores across the validation folds for brands (0.103). The FTDB had the highest mean F1 score for brands (0.815, SD 0.097), substantially outperforming the approach with the second-highest F1 score, the CRF (0.551, SD 0.037).

**Table 2:** Named entity recognition and benchmark validation for identifying brand and flavor mentions in vaping Instagram posts.

Approach			Value, mean (SD)				
			Precision		Recall		F1 score
**Nielsen scanner** **d** **ata match**							
	Brand	0.082 (0.061)		0.022 (0.015)		0.034 (0.024)	
	Flavor	0.507 (0.102)		0.417 (0.088)		0.446 (0.053)	
**Wikipedia** **d** **ata match**							
	Brand	0.224 (0.039)		0.102 (0.025)		0.140 (0.031)	
	Flavor	N/A^a^		N/A		N/A	
**CRF** ^b^							
	Brand	0.670 (0.080)		0.472 (0.044)		0.551 (0.037)	
	Flavor	0.695 (0.201)		0.496 (0.058)		0.573 (0.109)	
**RCNN** ^c^							
	Brand	0.797 (0.084)		0.405 (0.036)		0.535 (0.032)	
	Flavor	0.753 (0.232)		0.454 (0.082)		0.549 (0.109)	
**FTDB** ^d^							
	Brand	0.768 (0.095)		0.869 (0.103)		0.815 (0.097)	
	Flavor	0.860 (0.055)		0.801 (0.091)		0.828 (0.069)	

^a^N/A: not applicable.

^b^CRF: conditional random field.

^c^RCNN: residual convolutional neural network.

^d^FTDB: fine-tuned distilled bidirectional encoder representations from transformers.

### Flavors

For flavors, the mean precision was uniformly higher than the mean recall across all approaches. The flavor mean precision values ranged from 0.507 to 0.860, and the mean recall values ranged from 0.417 to 0.801. The FTDB exhibited the highest mean precision (0.860, SD 0.05), recall (0.801, SD 0.091), and F1 score (0.828, SD 0.069) for the flavor NER task. It also had the lowest SD of precision values across validation folds (0.055) when compared with the other approaches.

### Benchmark Comparisons

Overall, the machine learning NER models outperformed the benchmark matching approaches across the mean precision, recall, and F1 metrics, with comparable SDs. The major exception is the precision of the flavor mentions, which have greater variation in the RCNN and CRF NER models (RCNN: SD 0.232; CRF: SD 0.201) than the Nielsen scanner data matching (SD 0.102). However, the FTDB model exhibited the lowest SD of precision values for flavor mentions (SD 0.055), despite it also being a machine learning approach.

Comparing benchmarks, the larger Wikipedia data set performed better on brands for both precision (Nielsen: mean 0.082, SD 0.061; Wikipedia: mean 0.224, SD 0.039) and recall (Nielsen: mean 0.022, SD 0.015; Wikipedia: mean 0.102, SD 0.025). An ENDS product flavor list was not available from Wikipedia for comparison with the scanner data.

Table 3 compares the brands with the most mentions in our annotated Instagram data, using a 30% random test set of posts, with the top-selling brands in the Nielsen scanner data. Besides Puff Bar, there is little overlap between the top mentioned brands on Instagram and the top-selling brands identified through scanner data during the study period. Established brands comprising the largest market share of ENDS product sales (JUUL and Vuse) are not well-represented in our sample, which comprises emerging brands (Smok and GeekVape) and smaller niche brands (Majestea, a brand specializing in tea-flavored vape e-liquids and nicotine salts).

**Table 3:** Top brands in the Nielsen scanner and annotated Instagram data set by market share and brand mention share.

Brand		Share, n (%)
**Nielsen scanner (market; US $)**		
	JUUL	$330,397,506 (58.43)
	Vuse	$119,245,199 (21.09)
	Puff Bar	$38,852,586 (6.87)
	NJOY	$27,522,703 (4.87)
	Blu	$16,128,231 (2.85)
	Logic	$7,045,441 (1.25)
	Pop	$6,963,975 (1.23)
	Bidi Stick	$6,693,510 (1.18)
	ES	$2,982,509 (0.53)
	Jak	$2,292,140 (0.41)
**Instagram (mention; n=392)**		
	Smok	31 (7.9)
	GeekVape	24 (6.1)
	Puff Bar	16 (4.1)
	This is Salts	16 (4.1)
	Adore eLiquid	13 (3.3)
	Chief of Vapes	9 (2.3)
	Majestea	9 (2.3)
	Villain Vapors	9 (2.3)
	Conspiracy	6 (1.5)
	Orgnx	6 (1.5)

## Discussion

### Principal Findings

This study extends the ENDS literature by using computational methods to identify brand and flavor mentions on social media. Our findings suggest that NER models can improve upon a naïve approach of searching for known brands and flavors in noisy Instagram post text, both in terms of precision and recall. A notable finding is the superior performance of the FTDB model over all the approaches measured in this study. Although large increases in model performance are not uncommon when starting from pretrained models for computer vision and natural language processing tasks (see similarly large increases in performance on the WNUT17 NER data set in the *Comparison With Prior Works* section), the sizable leap in mean F1 scores between the FTDB (brands: 0.815; flavors: 0.828) when compared with the second best-performing CRF (brands: 0.551; flavors: 0.573) suggests that fine-tuning pretrained models for NER tasks on social media is worth the added complexity. In particular, the FTDB model far outperformed the other NER models in mean recall. Although all the NER models had reasonable precision (ie, terms predicted as brands or flavors were usually correct), the CRF and RCNN predicted a sizably smaller proportion of the true labeled brands and flavors in the validation sets than the FTDB.

The performance of the Wikipedia and Nielsen scanner brand dictionary look-up implies that a larger, more comprehensive list may improve the identification of known brands on Instagram. However, the many false positives may also suggest that some brand names are too generic when matched in isolation (eg, Carbon, Square, Epic, and Zoom). This may also indicate differences in coverage between popular brands in terms of sales and those being actively marketed and discussed on social media. The low level of overlap between the top-selling brands in the Nielsen scanner data and the most mentioned brands in our data set suggests that differing brand coverage may be a contributing factor. Given that current popular ENDS brands such as JUUL have been widely discussed on social media before the firm’s meteoric market growth [4], understanding ENDS brand engagement on social media may be an early indicator of emerging brands before they surface in sales data. Brands identified on social media should continually be cross-validated with other data sources, such as Nielsen, to better understand whether these models identify (1) emerging brands that will eventually become more established brands, (2) brands that will come and go, or (3) smaller-scale brands that are sold only on the web or in vapor shops.

### Comparison With Prior Work

Most published works on brand and flavor–named entity models come from research teams affiliated with large retail or e-commerce companies (eBay, Amazon, and Walmart) that use NER models to parse product titles or descriptions. These studies generally report precision and recall of >0.80 for brands and flavors [24,25,50,51]. However, despite the popularity of brand and flavor NER models on retail sites, there is considerably less published research on brand and flavor NER models for social media data. To our knowledge, the closest brand example is the WNUT17 data set [52] designed for benchmarking models to detect emerging named entities on social media (Twitter, Reddit, YouTube, and StackExchange). Although this data set does not contain a *brand* entity type, it does contain entities for *corporation* and *product*. Across 7 research teams, the original paper reported F1 scores on this task that ranged from 0.253 to 0.402 for detecting any instances of entity names and from 0.263 to 0.419 when only unique entity names were counted. More recently, newer models using fine-tuned transformer-based architectures have accounted for F1 scores in the range of 0.580 [53] to 0.600 [54]. This decline in NER performance on social media when compared with product titles and descriptions highlights the diversity of linguistic patterns and the use of unique syntactic conventions that make working with social media data challenging. Importantly, the authors note that *corporation* was generally a difficult class for systems to predict and speculate that this may be partly because of confusion between corporate and product entity types. For NER models with a flavor entity type, Xie et al [20] identified e-liquid flavors from posts on an e-cigarette forum. They reported an F1 score of 0.786, the lowest of all the 9 entity types they assessed. Although the reported metrics in this study are higher than those in the study by Derczynski et al [52], the test set for flavors in their study was comprised of only 13 observations, making it challenging to compare and draw strong generalization claims. Although direct comparisons with our models are complicated by differences in the sample composition, entity definitions, and social media platform, our best cross-validation results for brands (F1 score: mean 0.815, SD 0.097) outperform previous NER results on social media for entity types conceptually close to brands [52], and our results for ENDS flavors (F1 score: mean 0.828, SD 0.069) also outperform those previously reported in the literature [20].

### Implications

To predict the ENDS brands that may emerge and become popular among youths, there is a need to monitor social media platforms for ENDS advertising and conversations. An important component of this monitoring is to identify ENDS brands to be tracked and monitored on social media and other data sources (eg, scanner data) to better understand their marketing, appeal, and potential for growth to help the tobacco control community identify the next JUUL. Relying solely on traditional data sources such as Nielsen sales data or Kantar advertising expenditure data may not be adequate for helping to identify emerging brands. Indeed, new brands entering the marketplace may start by promoting their products on social media through organic posts (paid advertising of tobacco products is not allowed on social media platforms, including Facebook, Instagram, and Twitter) and selling products on the web as the barrier to entry is low. These brands would not appear in other traditional sales data sources; Nielsen data only captures brick-and-mortar retail sales and not web-based sales data. These data are also unlikely to show up in traditional advertising data sources, such as Kantar data, which only captures paid advertising and not organic social media posts. Brands that appear in sales and advertising data are likely to be more established brands spending money on advertising, distribution, and sales in retail stores.

### Limitations

Our study has several limitations. First, labeling brands is not always a straightforward task for human coders, especially when there is a need to distinguish between brands, products, and product lines. In addition, labeling flavors is complicated in the ENDS product space because of the presence of both *characterizing flavors* (flavors that describe existing food, beverages, or spices, such as grape, apple, and cinnamon) and *concept flavors* (ambiguous flavor names not tied to existing food, beverages, or spices, such as Bayou Blast and Midnight Madness) [55]. Difficulty in distinguishing between brand or corporation and product entities on social media [52] and difficulty in consistently identifying e-liquid flavors on social media [20] have been documented in the literature. Although we established sufficiently high intercoder reliability, our coders went through several rounds of calibration as there were disagreements about how to label certain mentions within posts. When there is ambiguity in how to label categories, or if labeling certain types of entities is inherently difficult, the accuracy of predictions for supervised machine learning models may decrease [56]. Second, most NER model metrics published in the literature report exact matches to the original labels, down to the character level. There may be cases where the model identified a ground-truth brand label; however, the predicted span was slightly offset, or only a portion of the full brand name was captured (eg, the model identified *BANG Bars*, but the annotation was *BANG Bars XL*). If partial matches are useful, the NER model evaluation metrics may understate the value of the model predictions. Although some works acknowledge this issue and incorporate partial match evaluation metrics into their reporting [57], this practice is not yet common in the literature. Third, as this study focused on a sample of Instagram posts, findings may not generalize beyond Instagram users to the general ENDS user population. Furthermore, although we theorized that the language used to describe ENDS brands and flavors in our study time frame is not characteristically different from other periods in the recent past, this hypothesis has not been tested and may benefit from further research. Finally, increasing the number of training examples could help improve model performance. Unlike more structured text examples for brand and flavor extraction, such as product titles, ENDS-related Instagram posts vary greatly stylistically, from marketing posts using concentrated bursts of product information to consumer posts that only mention vaping off hand. Although using transfer learning somewhat mitigates the need for more training data, having a larger corpus of example posts that better captures this diversity should improve model performance. In addition, methods for data augmentation [58] may also help improve performance.

### Future Work

Our results suggest several natural extensions for future work. First, as Instagram is a visual social medium, brand and flavor information is often conveyed in post images and text. Future work could use a multi-view learning approach [59] to incorporate information from both images and posts to improve model performance [60]. Longer-term NER models can be combined with entity linking [61] to update a database of established and emerging vaping brands and flavors. Entity linking, the process of reconciling extracted entities into a canonical entry, is often useful to combine with NER models because of the various ways brands and flavors can be mentioned in social media text (eg, extracted brand mentions of *puffbar*, *puff bar*, *puff bars*, and *p u f f b a r* should be associated with the canonical brand name *PuffBar*). This approach borrows the strengths of both the NER modeling and benchmark dictionary approaches outlined in this study. Although only brand and flavor NER models were examined in this work, extending to other named entities can help answer more nuanced research questions relevant to the tobacco control community. For example, NER results for relevant sentence subjects (brand or flavor mentions) could be linked to NER results for sentence objects relevant to public health outcomes, such as health claims [62] or adverse events [63,64]. NER can also be useful as a building block for more advanced downstream tasks, such as entity-based sentiment analysis [65] to better understand the sentiment associated with a given named entity (eg, the sentiment associated with certain brands or flavors). Finally, NER models could be used to support future infodemiology or infoveillance studies. Infodemiology [66,67] is an emerging science at the intersection of consumer and public health informatics that involves using publicly available data that can be identified, analyzed, and stored to provide important public health insights (eg, using web-based search query data to predict influenza outbreaks). For example, NER models could be used to identify and track ENDS brands or flavors over time to better understand whether their growth, or change in composition, is associated with related population health outcomes.

### Conclusions

This study demonstrates that NER models can be used to correctly identify ENDS brands and flavors in Instagram posts, with models showing favorable performance to previous research using NER to identify entities similar to brands and ENDS flavors in social media posts. The brands identified in our models had little overlap with brands from Nielsen scanner data, suggesting that these may be emerging brands. NER models may be a helpful approach for identifying emerging brands on social media before they appear in other data sources that capture more established brands. NER models address the challenges of manual brand identification being time consuming and difficult without pre-existing brand dictionaries, making them attractive for new brand identification. Brands identified on social media should continually be validated against other data sources, such as Nielsen scanner data, to help differentiate emerging brands that have become established from those with limited sales and distribution.

## Appendix

Multimedia Appendix 1 Query for identifying electronic nicotine delivery system Instagram posts.

## Abbreviations

BERT: bidirectional encoder representations from transformers
BFGS: Broyden–Fletcher–Goldfarb–Shannon
CRF: conditional random field
distilBERT: distilled bidirectional encoder representations from transformers
ENDS: electronic nicotine delivery system
FTDB: fine-tuned distilled bidirectional encoder representations from transformers
IO: inside–outside
NER: named entity recognition
RCNN: residual convolutional neural network
