# Supplementary material for: Identifying Electronic Nicotine Delivery System Brands and Flavors on Instagram: Natural Language Processing Analysis
Source: J Med Internet Res. 2022 Jan 18;24(1):e30257. doi: 10.2196/30257 (PMC8808345; doi:10.2196/30257)
Supplement: Multimedia Appendix 1 [file jmir_v24i1e30257_app1.docx]

Multimedia Appendix 1. Query for Identifying ENDS Instagram Posts

|  | Terms and Hashtags |
| --- | --- |
| Inclusion Terms | #vaping OR #vape OR #vapelife OR #vapecommunity OR #vapeon OR #vapeporn OR #vapefam OR #vapers OR #vapor OR #vaping101 OR #ecig OR #ecigs OR #ecigarettes OR #ecigdeals) |
| Exclusion Terms | (cannabis OR #skyporn OR #uk OR #420community OR #ukvapers OR cbd OR #cbd OR #cannabis OR #420 OR 420) |
